# Beverage-Specific Alcohol Sale and Cardiovascular Mortality in Russia

**DOI:** 10.1155/2010/253853

**Published:** 2011-01-23

**Authors:** Y. E. Razvodovsky

**Affiliations:** Central Scientific Laboratory, Grodno State Medical University, Grodno, 230009, Belarus

## Abstract

*Objective*. Recent research evidence suggests that the consumption of different types of alcoholic beverage may have a differential effect on cardiovascular diseases (CVD) mortality rates. The aim of this study was to examine the relation between the consumption of different beverage types and CVD mortality rates in Russia across the later-Soviet and post-Soviet periods. *Method*. Age-standardized male and female CVD mortality data for the period 1970–2005 and data on beverage-specific alcohol sales were obtained Russian State Statistical Committee (*Rosstat*). Time-series analytical modeling techniques (ARIMA) were used to examine the relation between the sales of different alcoholic beverages and CVD mortality rates. *Results*. Vodka consumption as measured by sales was significantly associated with both male and female CVD mortality rates: a 1 liter increase in vodka sales would result in a 5.3% increase in the male CVD mortality rate and a 3.7% increase in the female rate. The consumption of beer and wine were not associated with CVD mortality rates. *Conclusions*. The findings from this study suggest that public health efforts should focus on both reducing overall consumption and changing beverage preference away from distilled spirits in order to reduce cardiovascular mortality rates in Russia.

## 1. Introduction

High mortality from cardiovascular disease (CVD) in Russia and its profound fluctuations over the past decades have attracted considerable interest [1–3]. After a substantial decline in 1985–1988, coinciding with a major antialcohol campaign, CVD mortality rates was rapidly reversed and increased dramatically after dissolution of the USSR [4]. It was repeatedly emphasized that this phenomenon is only partially associated with the traditional CVD risk factors identified in epidemiologic studies [5, 6]. The results of population studies have shown that such risk factors as total cholesterol and apoprotein profile had little predictive value [7, 8]. This evidence suggests an influence of other powerful factors that are associated with increase in risk of cardiovascular death. Some scholars argue that these mortality fluctuations may be related to alcohol consumption [9, 10]. The findings suggest that population drinking and death rate from cardiovascular diseases are positively related phenomena in Russia. In particular, Nemtsov has reported a fairly close temporal covariation between trends in overall alcohol consumption and CVD mortality in Russia between 1965 and 1997 [10]. The results from recent time series analysis based on Russian data from 1959 to 1998 highlight that alcohol consumption has a positive and statistically significant association with both overall and premature male ischemic heart disease (IHD) mortality; a 1 litre change in per capita consumption was associated with a 3.6% increase in overall male IHD mortality and a 4.5% increase in the age group of 30–54 years [11]. This evidence challenged the view on a curvilinear relationship between alcohol and cardiovascular mortality that has been reported repeatedly in the literature [12–14]. 

Several studies point to binge drinking pattern as a potentially important contributor to higher cardiovascular mortality rate in Russia [15–17]. Using a pooled cross-sectional analysis, Gmel et al. [18] showed that in countries with favorable drinking pattern (e.g., France and Italy), per capita consumption was negatively associated with IHD mortality whereas a positive link was found in countries with the binge drinking pattern (e.g., Russia). The results from another study based on Russian data for the period from 1956 to 2005 suggest a positive association between fatal alcohol poisoning (as a proxy for binge drinking) and cardiovascular mortality rates [19]. 

Additional development of the idea that harmful drinking pattern can have detrimental effect on cardiovascular mortality in Russia came from individual level studies. For example, in the Novosibirsk cohort study was shown that frequent heavy drinking increased mortality from CVD [15]. Similarly, a case-control study of men aged 20–55 in the Udmurd Republic established that periods of heavy drinking were associated with an increased risk of CVD mortality [16]. This study reported that medium or greater level of intoxication occurred in a quarter of Russian men aged 20–55 dying from CVD. A study of 22658 forensic autopsies, performed in the Siberian city of Barnaul during 1990–2004, has shown that among autopsied men in the age 35–69 years who were reported to have died from cardiovascular diseases, 49% had ethanol detected in their blood and 14% had potentially lethal blood concentration of alcohol (4 g/L or more). Among autopsied women of the same age, 43% were BAC-positive, and in 13% the concentration was 4 g/L or more [20]. The role of excessive drinking pattern as a powerful risk factor for CVD mortality was emphasized in a study based on data from Moscow City that reported an increase in deaths from alcohol poisoning and cardiovascular disease on weekends [2]. These findings support the argument that binge drinking pattern may potentiate a negative role of alcohol as major cause of high cardiovascular death rate in Russia.

In fact, such important aspect of drinking as beverage preference should be considered in order to clarify the role of drinking pattern in alcohol-cardiovascular mortality relations. There is suggestive evidence that alcoholic beverage preference is associated with drinking pattern. For example, in Russia occasional consumption of strong spirits (vodka) in big doses is a long-standing tradition [21]. Furthermore, in Russia, binge drinking men and women are almost exclusively vodka drinkers while light to moderate drinkers consumed a much wider variety of beverages [10]. It was shown, however, that consumption of spirits per capita is positive related to heart disease mortality in six of the nine Western countries [22]. Similarly, it was highlighted that in Belarus relationship between alcohol and CVD mortality was stronger for consumption of spirits (vodka) relative to overall alcohol as well as wine/beer consumption [23]. In line with these findings, we assume that if occasional heavy drinking of spirits increases the risk of cardiovascular death, countries where this is a predominant drinking pattern should display positive association between vodka sales and CVD mortality at the aggregate level. To test this hypothesis trends in beverage-specific alcohol sales per capita and CVD mortality rates from 1970 to 2005 in Russia were analyzed applying ARIMA time series analyses. 

## 2. Material and Methods

### 2.1. Data

The data on age-adjusted sex-specific cardiovascular mortality rates per 1000,000 of the population are taken from the Russian vital statistics registration system. The Goscomstat's cause of death classification has undergone several changes in recent decades. Until 1988, the cause of death classification was based upon the Soviet nomenclature which had a limited number of causes of death in comparison with the International Classification of Diseases (ICD) system. From 1989–1998, Rosstat used a coding scheme that was based on ICD-9. From 1999, a new coding system based on ICD-10 was introduced. Rosstat issued a table of correspondence between its classification system and ICD-9 and ICD-10 and it has been claimed that the Russian system of coding was and is compatible with the ICD. For example, Rosstat's code 84–102 (1989–1998) “Diseases of circulatory system” corresponds with ICD-9 code E 390–E 459 and code 115–147 (since 1999) corresponds with ICD-10 code X 100-X 199. The data on per capita beverage specific alcohol sales were taken from Rosstat's annual reports.

### 2.2. Statistical Analysis

To examine the relation between changes in the consumption of different types of alcoholic beverage and CVD mortality across the study period, a time-series analysis was performed using the statistical package “Statistica.” Bivariate correlations between the raw data from two time-series can often be spurious due to common sources in the trends and due to autocorrelation [24]. One way to reduce the risk of obtaining a spurious relation between two variables that have common trends is to remove these trends by means of a “differencing” procedure, as expressed in the following formula:

(1) $$\nabla x_{t}=x_{t}-x_{t-1}.$$

This means that the annual changes “∇” in variable “*X*” are analyzed rather than raw data. The process whereby systematic variation within a time series is eliminated before the examination of potential causal relationships is referred to as “prewhitening.” This is subsequently followed an inspection of the cross-correlation function in order to estimate the association between the two prewhitened time series. It was Box and Jenkins [25] who first proposed this particular method for undertaking a time series analysis and it is commonly referred to as ARIMA (autoregressive integrated moving average) modeling. We used this model specification to estimate the relationship between the time series CVD mortality rate and beverage-specific alcohol sales in this paper. In line with previous aggregate studies [11, 24], we estimated semilogarithmic models with logged output. The following model was estimated:

(2) $$\nabla LnM_{t}=a+\beta \nabla A_{t}+\nabla N_{t},$$

where ∇ means that the series is differenced, *M* is CVD mortality rates, *a* indicates the possible trend in CVD mortality due to other factors than those included in the model, *A* is the alcohol sale, *β* is the estimated regression parameter, and *N* is the noise term. The percentage increase in CVD mortality rate associated with a 1-litre increase in alcohol sale is given by the expression: (exp (*β*_1_) − 1)∗100.

## 3. Results

**Figure 1:**
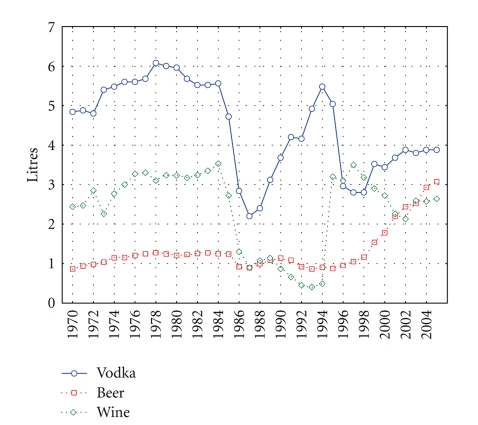
Beverage-specific alcohol sales in Russia between 1970 and 2005.

Descriptive statistics are presented in Table 1. Across the whole period, the male CVD mortality rate was 1.5 times higher than the female rate (11714.5 versus 7823.3 per 1,000,000). The average per capita alcohol consumption figure was 8.2 liters with vodka being the drink overwhelmingly consumed. However, these mean figures mask differing trends among the beverages across the period (Figure 1). While there has been a slight drop in vodka sales from 4.84 liters in 1970 to 3.88 liters in 2005 and wine sales have remained at roughly the same level, there has been a sharp growth in beer sales—especially in recent years. Between 1998 and 2005, the per capita consumption figure for beer rose from 1.16 to 3.08 liters. It is also worth noting that beverage sales have experienced sharp fluctuations across the period. Thus, an especially sharp fall was recorded in vodka and wine sales in 1984–1987 that coincided with Mikhail Gorbachev's antialcohol campaign (Figure 1). Similarly, the collapse of the Soviet Union and the ending of the state's alcohol monopoly in the early 1990s was accompanied by a sharp rise in vodka sales.

**Figure 2:**
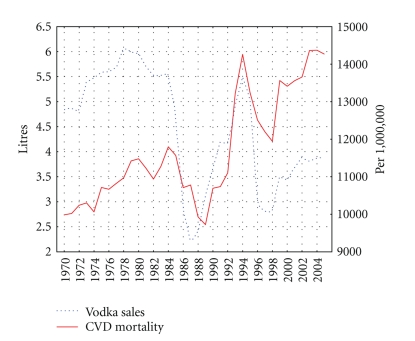
Trends in male CVD mortality rate (right scale) and vodka sales per capita (left scale) in Russia between 1970 and 2005.

**Figure 3:**
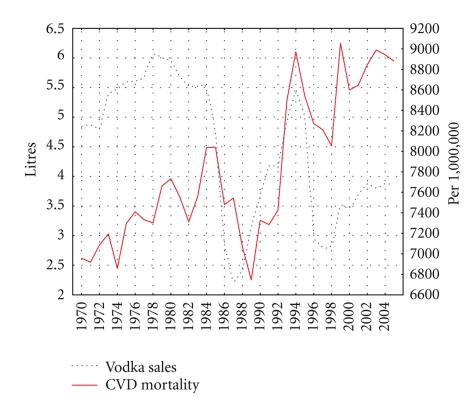
Trends in female CVD mortality rate (right scale) and vodka sales per capita (left scale) in Russia between 1970 and 2005.

The trends in the age-adjusted, sex-specific CVD mortality rates are displayed in Figures 2 and 3. For both sexes, the time series CVD mortality rates fluctuated greatly over the period: increasing steadily from 1970 to 1984 before dropping sharply in the years 1984–1986. From 1988-1989 the series again started on an upward trend, before jumping dramatically during 1991 to 1994. From 1995–1998, there was a fall in the rates before they again began to rise while a decrease in rates has been recorded in the most recent years.

As can be seen from Figures 1 and 2, there were sharp trends in the time series data across the study period. These trends were removed by means of a first-order differencing procedure. After prewhitening the cross-correlations between beverage-specific alcohol sales and the CVD mortality, time series were inspected. This indicated that there was a statistically significant cross-correlation between vodka sales and CVD mortality for males and females at lag 0 (Tables 2-3). At the same time, there is no cross-correlation between the level of wine/beer sales and CVD mortality rates (Tables 2-3). The specification of the bivariate ARIMA model and outcome of the analyses are presented in Table 4. The estimated effects of vodka sales on the CVD mortality rate are clearly statistically significant for both sexes—as a 1 liter increase in vodka sales would result in a 5.3% increase in the male CVD mortality rate and in 3.7% increase in female CVD mortality rate.

## 4. Discussion

The dramatic CVD mortality fluctuations in Russia during the last decades are unprecedented in an industrialized country. These trends coincide with major political events: the antialcohol campaign in the mid-1980s, the political and economic transition following the breakup of the Soviet Union in 1991, and the worsening economic situation associated with the financial crisis in 1998. The graphical evidence suggests that spirits drinking and mortality rates are positively related phenomena in Russia: Gorbachev's antialcohol campaign 1985–1988 was associated with a rapid reduction in the level of vodka sales per capita and cardiovascular mortality rates, while increasing vodka sale in the transitional period has been linked to higher mortality rates. The coincident trends between the level of vodka sales and CVD mortality in the mid-1980s indicate that a restriction of vodka availability can be considered as an effective measure of mortality prevention. 

The finding that of the three beverages vodka alone was associated with CVD mortality in Russia seemingly supports earlier research which has suggested that beverage-specific effects are strongest in cultures in which a heavy episodic drinking of spirits predominates [9, 18, 23, 26]. This would certainly seem to be the case in Russia as despite the recent growth in the consumption of beer, vodka continues to be the beverage that dominates in terms of consumption. 

It is important to point out, that the time series analysis suggests a positive relationship between vodka sale and CVD mortality, time series at lag 0. It means that independent variable is directly influencing the dependent variable and that there is no evidence of a lagged relationship between the two time series. In fact, the contemporaneous association between the two variables may support the point that binge drinking of vodka is a risk factor for CVD mortality. 

There is a vide range of pathological mechanisms by which binge drinking of vodka can lead to death from cardiovascular disease. It was shown, for example, that low density lipoproteins are increased by heavy drinking episodes, resulting in detrimental effects on the cardiovascular system [27]. Further, irregular heavy drinking predisposes to abnormality of the myocardial conducting system and to a reduction in the threshold for arrhythmia, especially atrial fibrillation [28]. Episodic heavy drinking also has adverse effects on blood clotting [27, 29]. 

Before concluding, several potential limitations of this study must be mentioned. In particular, we relied on official alcohol sales data as a proxy measure for trends in alcohol consumption across the period. However, the unrecorded consumption of alcohol was commonplace in Russia throughout the study period, especially in the mid-1990s, when a considerable proportion of vodka came from illicit sources [30, 31]. Further, there was also the risk of omitted variable bias in this work. It should be emphasized that vodka sale per capita before the start of antialcohol campaign exceeded the level recorded in the early 1990s, while CVD mortality rates in the early 1990s exceeded the level of early 1980s significantly. This means that some additional factors came into play in the yearly 1990s. 

It seems plausible that CVD mortality crisis in the mid-1990s was to a great extent due to changed alcohol consumption structure, when 80% of all alcohol in Russia was consumed in the form of spirits. It is obvious that wine became less available, compared to vodka in Russia during antialcohol campaign with the destroying most of the vineyards across the country and then the break-up of the Soviet Union cutting off wine supplies from Moldova, Ukraine, Georgia, and other former Soviet republics [31]. Another potential reason for the detrimental effect of alcohol was deterioration of his quality. According to the data from the State Statistics Service, up to 45% of vodka sold during that period did not meet quality standards [10]. 

Some researchers also believes, that the dramatic increase in CVD mortality in 1990s was driven by the psychosocial distress of economic and political reforms and facilitated by the availability of vodka after abolishing the state alcohol monopoly in January 1992 [32]. The collapse of the USSR in 1991 and growth of consumer prices in 1992 were followed by declining of living standards of the majority of population and deterioration of public health [33, 34]. The impact of acute socioeconomic transition has been exacerbated by a lack of social cohesion, erosion of social capital, and rising income inequality [35, 36]. It should be noted, however, that the increase in mortality between 1991 and 1994 reached its peak for people of working age, that is, the main alcohol consumers [33, 35]. Moreover, there is assumption that most deceased were hard drinkers [31].

In conclusion, the present study replicates previous findings from other settings that suggest that CVD mortality tends to be more responsive to changes in spirits consumption per capita than to the wine/beer consumption. Assuming that drinking vodka is usually associated with intoxication episodes, these findings provide additional evidence that substantial proportion of cardiovascular deaths in Russia are due to acute effect of binge drinking. The findings from the present study have important implications as regards alcohol policy in Russia suggesting that any attempts to reduce overall consumption should also be linked with efforts through differential taxation to shift beverage preference away from spirits.

## Figures and Tables

**Table 1:** Descriptive statistics, Russia 1970–2005.

	Mean	Std. Dev.
Total alcohol sales (liters)	8.18	2.03
Vodka sales (liters)	4.44	1.15
Wine sales (liters)	2.39	0.99
Beer sales (liters)	1.33	0.58
CVD mortality rate males (per 1,000,000)	11714.5	1462.2
CVD mortality rate females (per 1,000,000)	7823.3	713.2

**Table 2:** The results of cross-correlation analysis between prewhitened time series for males. Effects of beverage-specific alcohol sale per capita on CVD mortality rate.

	Vodka sales		Wine sales		Beer sales	
Lag	*r*	SE	*r*	SE	*r*	SE
−3	−0.124	0.177	−0.188	0.176	−0.052	0.177
−2	0.231	0.174	−0.306	0.174	−0.125	0.174
−1	0.264	0.172	−0.257	0.172	−0.187	0.172
0	0.594	0.167	−0.106	0.169	−0.087	0.169
1	0.273	0.172	0.1206	0.172	−0.077	0.172
2	−0.231	0.174	0.126	0.174	−0.152	0.174
3	−0.143	0.177	0.186	0.177	−0.236	0.177

**Table 3:** The results of cross-correlation analysis between prewhitened time series for females. Effects of beverage-specific alcohol sale per capita on CVD mortality rates.

Lag	Vodka sales		Wine sales		Beer sales	
	*r*	SE	*r*	SE	*r*	SE
−3	−0.008	0.177	−0.138	0.174	−0.059	0.177
−2	−0.123	0.174	−0.170	0.174	−0.143	0.174
−1	−0.165	0.172	−0.266	0.172	−0.216	0.172
0	0.493	0.169	−0.052	0.169	−0.012	0.169
1	0.131	0.172	0.115	0.172	−0.021	0.172
2	−0.212	0.174	0.236	0.174	−0.140	0.174
3	−0.207	0.177	0.149	0.177	−0.157	0.177

**Table 4:** Estimated effects (bivariate ARIMA model) of vodka sales on CVD mortality rates.

Parameter	Model	Estim.	St. Error	*t*	*p*
CVD mort. males	0,1,0*	0,053	0,012	4,076	0,000
CVD mort. females	0,1,0	0,037	0,12	3,129	0,004

*The general form of nonseasonal ARIMA model is (*p*, *d*, *q*p), where *p* is the order of the autoregressive parameter, *d*—the order of differencing, and *q* is the order of the moving average parameter. *Q* test for residuals are satisfactory in all models.
